# CTRP5-Overexpression Attenuated Ischemia-Reperfusion Associated Heart Injuries and Improved Infarction Induced Heart Failure

**DOI:** 10.3389/fphar.2020.603322

**Published:** 2020-12-22

**Authors:** Meng Peng, Yuan Liu, Xiang-qin Zhang, Ya-wei Xu, Yin-tao Zhao, Hai-bo Yang

**Affiliations:** Department of Cardiology, The First Affiliated Hospital of Zhengzhou University, Zhengzhou, China

**Keywords:** CTPR5, heart, ischemia/reperfusion, myocardial infarction, heart failure

## Abstract

**Aims:** C1q/tumor necrosis factor (TNF)-related protein 5 (CTRP5) belongs to the C1q/TNF-α related protein family and regulates glucose, lipid metabolism, and inflammation production. However, the roles of CTRP5 in ischemia/reperfusion (I/R) associated with cardiac injuries and heart failure (HF) needs to be elaborated. This study aimed to investigate the roles of CTRP5 in I/R associated cardiac injuries and heart failure.

**Materials and Methods:** Adeno-associated virus serum type 9 （AAV9）vectors were established for CTRP5 overexpression in a mouse heart (AAV9-CTRP5 mouse). AAV9-CTRP5, AMPKα2 global knock out （AMPKα2^−/−^）and AAV9-CTRP5+ AMPKα2^−/−^ mice were used to establish cardiac I/R or infarction associated HF models to investigate the roles and mechanisms of CTRP5 *in vivo*. Isolated neonatal rat cardiomyocytes (NRCMS) transfected with or without CTRP5 adenovirus were used to establish a hypoxia/reoxygenation (H/O) model to study the roles and mechanisms of CTRP5 *in vitro*.

**Key Findings:** CTRP5 was up-regulated after MI but was quickly down-regulated. CTRP5 overexpression significantly decreased I/R induced IA/AAR and cardiomyocyte apoptosis, and attenuated infarction area, and improved cardiac functions. Mechanistically, CTRP5 overexpression markedly increased AMPKα2 and ACC phosphorylation and PGC1-α expression but inhibited mTORC1 phosphorylation. In *in vitro* experiments, CTRP5 overexpression could also enhance AMPKα2 and ACC phosphorylation and protect against H/O induced cardiomyocytes apoptosis. Finally, we showed that CTPR5 overexpression could not protect against I/R associated cardiac injuries and HF in AMPKα2^−/−^ mice.

**Significance:** CTRP5 overexpression protected against I/R induced mouse cardiac injuries and attenuated myocardial infarction induced cardiac dysfunction by activating the AMPKαsignaling pathway.

## Introduction

Myocardial infarction (MI) is one of the most life-threatening diseases worldwide (Reed et al., 2017). Although myocardial reperfusion is effective and beneficial in deQcreasing mortality and improving the survival rate of patients with MI (Reed et al., 2017), reperfusion could also lead to cardiac injuries by causing cardiomyocyte death with complex mechanisms (Hausenloy and Yellon, 2016). It is very important to clarify the precise mechanisms and to explore new strategies for preventing I/R and MI associated heart failure.

AMP-activated protein kinase (AMPK), the most important kinase for regulating glucose and fatty acid oxidation for producing ATP, is a critical regulator of the adaptive response to cardiomyocyte stress occurring in myocardial I/R and MI (Qi and Young, 2015; Daskalopoulos et al., 2016). Activated AMPK maintains ionic gradients for electrical excitability and myofibrillar contractile by supplying ATP. AMPK also reduces endoplasmic reticulum stress, prevents oxygen species generation, and inhibits mitochondria permeability transition pore opening for preventing cardiomyocyte apoptosis (Qi and Young, 2015; Daskalopoulos et al., 2016). Investigations have demonstrated that AMPK was transiently up-regulated after ischemia and rapidly fallen to baseline or even below the baseline (Qi and Young, 2015; Daskalopoulos et al., 2016). AMPK inhibition or deletion has been demonstrated to exacerbate pathological stress induced cardiomyocyte apoptosis, which finally results in cardiac dysfunction (Wang et al., 2012). AMPK activation by metformin, melatonin, and Tanshinone IIA could improve ischemia-induced cardiac dysfunction by inhibiting cardiomyocyte apoptosis and blocking the expression of key apoptotic proteins, including B cell lymphoma-2 associated X (BAX) and cleaved caspase-3 (c-caspase 3) (Nesti and Natali, 2017; Yu et al., 2017; Zhang et al., 2019). Therefore, activating AMPK could be a potential strategy for ameliorating I/R and MI injury.

C1q/TNF-related proteins (CTRPs) family, have been recognized as a paralog of adiponectin and might possess similar biological functions as adiponectin since they have similar modular organizational structures (Si et al., 2020). Adiponectin has shown benefits for metabolic regulation as it modulates glucose production and fatty acid oxidation (Wang and Scherer, 2016). Adiponectin could also protect against cell apoptosis and reduce inflammation through receptor-mediated AMPK signaling regulation (Wang and Scherer, 2016). Adiponectin administration diminished I/R induced infarct size and cardiomyocyte apoptosis via an AMPK dependent pathway (Shibata et al., 2005). Some previous studies have demonstrated that CTRP family members, including CTPR3, CTRP8, and CTPR9, regulate pathological cardiac remodeling with a similar biological mechanism compared with adiponectin. CTRP3 attenuates streptozotocin induced diabetic cardiomyopathy by activating AMPKα via the cAMP/mitogen-activated protein kinase pathway (Ma et al., 2017). CTRP9 administration reduced I/R associated cardiomyocyte apoptosis and infarct size by increasing AMPK phosphorylation (Kambara et al., 2012).

CTRP5, another member of the CTRP family, also participates in metabolism regulation and regulates the development and progression of coronary artery disease (Si et al., 2020). Furthermore, Park et al. (2009) demonstrated that CTRP5 might be a potential biomarker of mitochondrial dysfunction and a potent regulator for glucose uptake and fatty acid oxidation via regulating AMPK activity. However, to date, the role and mechanisms of CTR5 in regulating ischemia associated cardiac remodeling and heart failure have not been explored. This study investigated the role of CTRP5 in I/R and explored whether CTRP5 overexpression could alleviate ischemia associated heart failure *in vivo* and *in vitro*.

## Materials and Methods

### Animal Experiments

All animal care and experimental protocols were performed according to the Guidelines for the Care and Use of Laboratory Animals of Zhengzhou University and were approved by the Ethics Committee of Zhengzhou University. All animal-based experimental procedures complied with the Care and Laboratory animal guidelines published by the United States National Institutes of Health (NIH publication, revised 2011).

### Generation of Transgenic Mice for CTRP5 Overexpressing

#### AAV Generation and Injection

An open reading frame of mouse CTRP5 (NM_001278431) was cloned into the pZac2.1 vector (under control of an α-MHC promotor) and was subsequently used for AAV generation. AAVs were generated by the AAV Vectors. Adeno-associated virus vector expressing human CTRP5 (AAV-CTRP5) or green fluorescent protein (AAV-GFP) were injected into mice via the jugular vein with 1 × 10^12^ viral genome particles per mouse (Shin et al., 2011). After AAV-CTRP5 or AAV-GFP injection for 3 weeks, mice were used for the following experiments.

#### Mouse Model of Ischemia-Reperfusion Injury

An ischemia-reperfusion injury mouse model was established according to the published protocol (Kataoka et al., 2014). Briefly, mice were first anesthetized and intubated. Then, the left anterior descending coronary artery (LAD) was isolated for ligation with a suture and a snare occluder. After 60 min of ischemia, the snare occluder was loosened for reperfusion. The LAD was retied after 24 h of reperfusion. Evans blue was systemically injected into mice for examining the non-ischemic tissue. Mice hearts were excised and incubated with 2,3,5-triphenyltetrazolium chloride to show the infarction area. The left ventricular area (LV), the area at risk (AAR), and the infarct area (IA) were quantified with Image Pro 6 software.

#### Mouse Model of Myocardial Infarction

To induce myocardial infarction, the LAD was ligated for 4 weeks without loosening. Echocardiography was performed to evaluate mouse cardiac function before sacrifice.

### Echocardiographic Analysis

Transthoracic echocardiography (VeVo 2100 Imaging System equipped with 15-MHz probe) was performed to assess cardiac structure and function after 4 weeks of myocardial infarction. Mice were anesthetized by inhaling 1% isoflurane before cardiac function assessment. A short axis view at the papillary muscle level with M mode was obtained for measurements. Left ventricular internal diameter at diastole (LVID; d), LV end internal diameter at systole (LVID; s), ejection fraction (EF), and fractional shortening (FS) were detected and calculated for at least five cardiac cycles consecutively in 2D M-mode mode tracing. The calculation package was, as follows: %EF (M-Mode) = 100*[(LV Vol; d- LV Vol; s)/LV Vol; d]; %FS (M-Mode) = 100*[(LVID; d- LVID; s)/LVID; d]. LV Vol; d (M-Mode) = [7/(2.4 + LVID; d)] LVID; d^3^; LV Vol; s (M-Mode) = [7/(2.4 + LVID; s)] LVID; s^3^.

### Neonatal Rat Cardiomyocyte Isolation

Neonatal rat cardiomyocytes (NRCMSs) were isolated according to published protocols (Tao et al., 2018) and were cultured in DMEM supplemented with 10% fetal calf serum. Briefly, Neonatal rats (0–3 days, Sprague-Dawley rats) were first anesthetized with carbon dioxide and sacrificed by cervical dislocation. The ventricular tissues were removed and then minced into 1 mm^3^ pieces. Minced ventricular tissues were located in a 200 ml glass bottle and digested into single cell suspension by adding trypsin collagenase 2. The cell suspension was collected and fetal calf serum was added to eliminate the enzyme activity. After being filtered and centrifuged, the resuspended cell suspension was layered on the top of the percoll solution for standard protocols of acceleration and deceleration speed 0. NRCMSs was isolated from the newly formed layer between the percoll solutions. NCRMs were cultured on gelatin-coated six-well plates with a density of 2 × 10^5^/ml and placed in a humidified incubator at 37°C and 5% CO_2_.

NRCMSs were transfected with adenovirus vector encoding CTRP5 (Ad-CTRP5) or Ad-GFP (as a control) at a multiplicity of infection of 20 for 24 h. The Anaero Pack System (Mitsubishi GAS Chemical Co., Inc.) was used to establish the hypoxia/reoxygenation cellular model. Briefly, NRCMs were cultured in Dulbecco’s modified Eagle’s medium (DMEM), added with 10% fetal bovine serum (FBS) at 37°C with 95% air and 5% CO_2_ for adenovirus infection or compound C (10 μM) treatment. The prepared NRCMs were then exposed to an anaerobic medium (serum and glucose free) in Anaero Pack System (providing an anoxic mixture gas: 95% N_2_ and 5% CO_2_) for 2, 4 or 6 h at 37°C. After the hypoxia incubation, the NRCMs were allowed for reoxygenation for 6 or 12 h in a fresh culture medium at 37°C with 95% air and 5% CO_2_.

### Caspase 3 Activity Examination

Ac-DEVD-AFC Caspase-3 Fluorogenic substrate kit (BD Pharmingen) was used for testing the caspase-3 activity according to the manufacture’s instructions. Briefly, heat tissue or cardiomyocytes were lysed on ice for collecting the supernatant. After protein quantification, 50 µg proteins were added into the assay buffer containing 10 mM dithiothreitol (DTT). The 7-amno-4-trifluoromethylcoumarin (AFC) could be monitored in a microplate spectrophotometer (Molecular Devices) at an excitation wavelength of 400 nm and an emission wave length of 520 nm.

### Western-Blots Analysis

Mouse left ventricle tissue or NRCMS were collected and lysed. BCA Protein Assay kit (Thermo Fisher Scientific) was used for quantifying protein concentrations. Fifty micrograms protein of each sample was separated by electrophoresis and transferred to polyvinylidene fluoride membranes. The bots were blocked with 5% nonfat milk dissolved in TBST buffer for 1 h and then were incubated with primary antibodies overnight at 4°C. The next day, blots were washed with TBST buffer and then were incubated with appropriate secondary antibodies (1:2,000) at room temperature for 60 min. Blots were examined by Bio-Rad Imaging System (Bio-rad), and the results were normalized to β-actin for relative quantitative analysis.

### Real-Time Quantitative Polymerase Chain Reaction (RT-PCR) Analysis

Total RNA was prepared using TRIZOL reagent (Invitrogen, CA, United States). The cDNA was prepared from 2 μg of total RNA using the first strand complementary DNA synthesis kit (Invitrogen, CA, United States) according to the manufacturer’s instruction. A 20 μl reaction system (containing 1 μl cDNA, 1 μl primers, 10 μl SYBR green dye, and 8 μl H2O). A LightCycler 2.0 system (Roche, Basel, Switzerland) was used to detect the PCR amplification of the prepared reaction system. The primers used in this study are shown in Supplementary Table S1.

### TUNEL Staining for Apoptosis Determination

Terminal deoxynucleotidyl-transferase-mediated dUTP nick end labeling (TUNEL) staining was performed to determine cellular apoptosis by using a TUNEL detection kit (Roche) according to the manufacture’s instruction. Briefly, paraffin sections of mouse heart were dewaxed, hydrated, and then incubated with Proteinase K for 20 min at room temperature. Fifty microliters TUNEL reaction mixture (100 μl TdT + 900 μl fluorescein-labeled dUTP) was prepared and added on top of each specimen. The negative control group was only managed with 50 μl fluorescein-labeled dUTP, and the positive control group with another 100 μl DNase 1 before adding 50 μl TUNEL reaction mixture. After incubation with reaction mixture at 37°C for 1 h, specimens were incubated with 50 μl converter-POD for 30 min at 37°C and then incubated with 50 μl IDAB substrate for 10 min at room temperature respectively. Finally, hematoxylin was used to label the nucleus. Image Pro 6.0 was used to analyze pictures.

### LDH Examination

NRCMSs were seeded in six-well plates and were transfected with Ad-CTRP5 or Ad-GFP (as a control) at a 20 MOI for 24 h or were treated with compound (10 μM) for inhibiting AMPK activity. NRCMSs were incubated in H/R conditions as previously mentioned. The LDH leakage was calculated by testing the release of LDH from NRCMSs using an LDH detection Kit (Beyotime, Jiansu, China) according to the manufacturer’s instructions.

### ATP Content Measurement in Heart Tissue

Heart tissue ATP content was measured using the Enhanced ATP Assay Kit (Beyotime, Jiansu, China) according to the manufacturer’s instructions. Briefly, 20 mg heart tissue was added with 200 μl of ATP lysate buffer and then was fully homogenized to ensure that the heart tissue was completely cracked. The heart tissue lysate was centrifuged at 4°C with 12,000g for 5 min to collect the supernatant for subsequent ATP content determination according to the manufacturer’s instructions.

### Statistical Analysis

Where applicable, the collection and analysis of data were blinded for *in vivo* and *in vitro* experiments. All data were exhibited as the means ± SEM. Student’s *t* test for unpaired data was used for comparison between the two groups. One-way ANOVA followed by *post hoc* test was performed to compare the significance among multiple groups. If the variance was uniform, S-N-K (Student-Newman-Keuls) was used for test comparison among different groups. If the variance had a significant difference, Dunnett’s T3 was used for test comparison among different groups. Data analysis was performed with SPSS 19.0. *p* < 0.05 was defined as statistically significant.

## Results

### Ischemia or Hypoxia-Reoxygenation Associated CTRP5 Expression *In Vivo* and *In Vitro*

**FIGURE 1:**
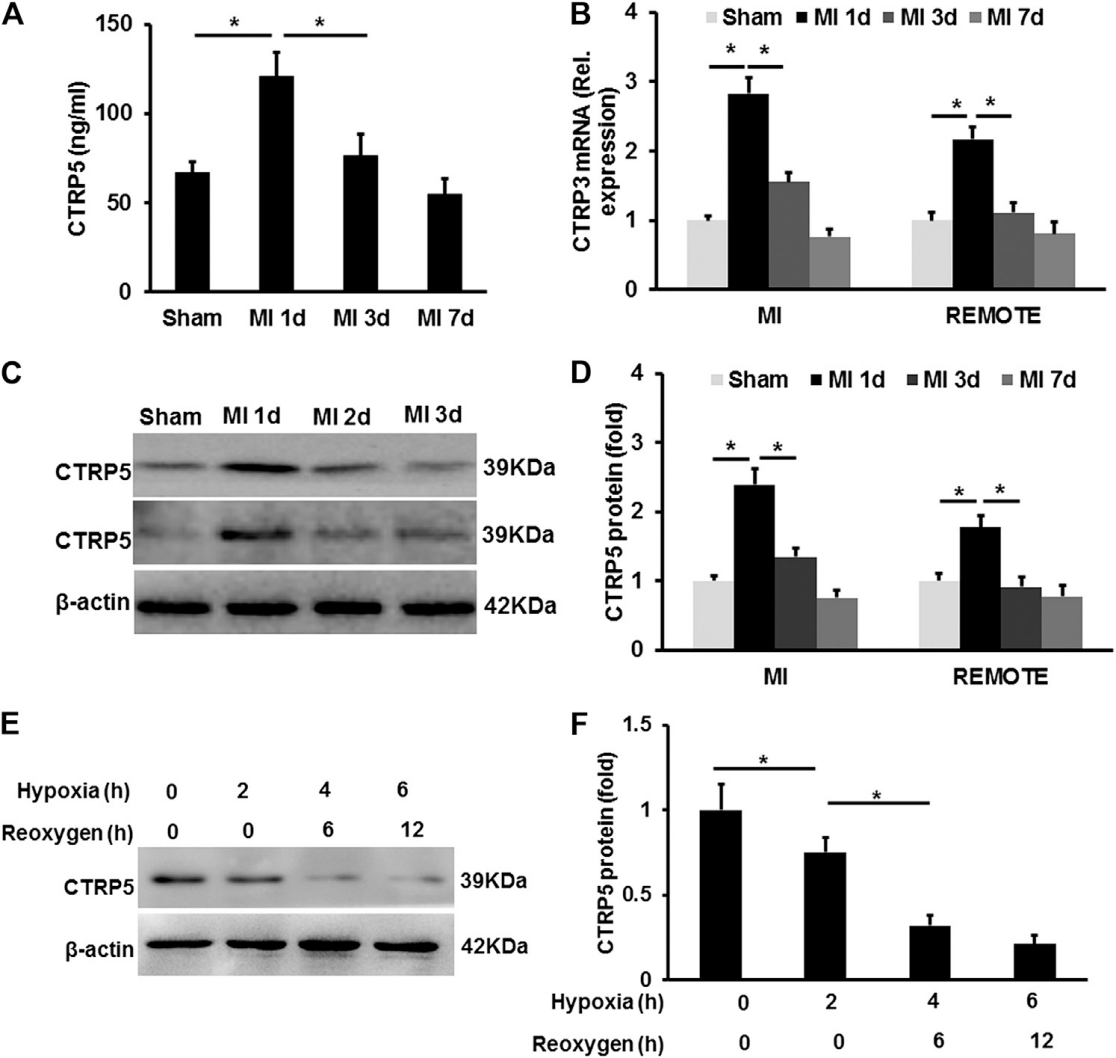
C1q/TNF-related protein 5 (CTRP5) expression in mouse heart and eonatal rat cardiomyocytes (NRCMSs) under pathophysiological condition **(A)** plasma CTRP5 level after myocardial infarction (MI) or sham surgery at different time points, which was quantified by Elisa analysis (*N* = 7–10); **(B)** mRNA expression of CTRP5 in mouse heart after MI or sham surgery at different time points (*N* = 6); **(C,D)** protein expression of CTRP5 in mouse heart after MI or sham surgery different time points (*N* = 6); **(E,F)** protein expression of CTRP5 in NRCMSs after hypoxia for 2, 4 or 6 h firstly and then reoxygenation for 6 and 12 h. Relative protein level of CTRP5 was calculated by ImageJ. Data are shown as mean ± SEM. *p < 0.05 represented the difference between the two groups connected by line was statistically significant.

CTRP5 was up-regulated in mouse serum after MI, but it was down-regulated after 1 day of MI and even lower compared with the control group 7 days after MI (Figure 1). CTRP5 expression at mRNA level was significantly up-regulated at both the MI area and remote area 1 day after MI and then declined (Figure 1). Similarly, the protein expression of CTRP5 was increased both at MI and remote area 1 day after MI and was significantly down-regulated as time went on (Figure 1). In isolated NRCMS, hypoxia-reoxygenation induced CTRP5 down-regulation (Figure 1). These results implied that CTRP5 might involve in regulating ischemia associated heart injuries.

### CTRP5 Overexpression Attenuated Ischemia-Reperfusion Induced Mouse Heart Injuries

**FIGURE 2:**
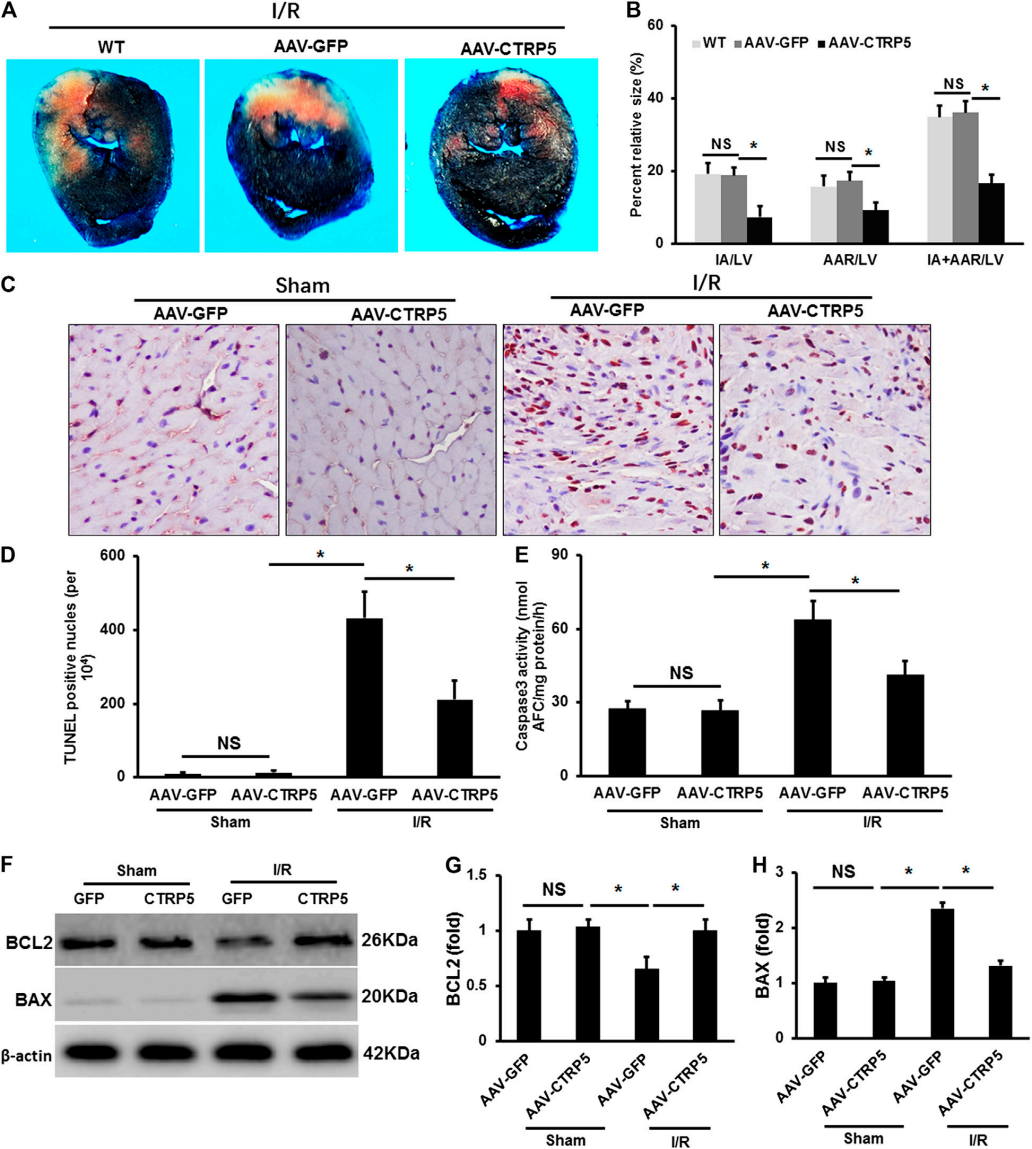
Adeno-associated virus serum type 9 (AAV9) mediated CTRP5 overexpression attenuated ischemia/reperfusion (I/R) associated injuries in mouse heart. **(A)** CTRP5 overexpression reduced I/R induced infarction size. C57B6/L mice were systemically administrated with an AAV vector to express GFP (AAV-GFP, 4.0 × 10^7^ PFU) or human CTRP5 (AAV-CTRP5, 4.0 × 10^7^ PFU) for 3 weeks and then were subjected to I/R surgery. Heart sections were stained with Evans blue and 2,3,5-triphenyltetrazolium chloride (TTC) after I/R. **(B)** Quantitative analysis of infarct area (IA, white)/left ventricle area (IA/LV), area at risk (AAR, red)/LV and (IA+AAR)/LV ratios (*N* = 7–10). **(C,D)** Representative pictures of TUNEL staining and quantitative analysis for apoptotic nuclei at 24 h after I/R (*N* = 7–10). **(E)** Caspase 3 activity analysis (*N* = 6). **(F–H)** Blots and relative quantitative analysis of Bax and Bcl2 expression (*N* = 6, normalized to β-actin). Data are shown as mean ± SEM. *p < 0.05 represented the difference between the two groups connected by line was statistically significant.

CTRP5 were overexpressed successfully in mouse heart after 3 weeks of AAV9 vein injection (Figure 2). Mice with or without CTRP5 overexpression were subjected to 60 min of myocardial ischemia and 24 h of reperfusion. As shown in Figure 2B, CTRP 5 significantly reduced the mean infarct area/left ventricle area (IA/LV), area at risk/LV (AAR/LV) and (IA+AAR)/LV ratios compared to control group (AAV-GFP), no significance of these three ratios was tested between WT and AAV-GFP group. The apoptosis of cardiomyocytes aggravated after I/R management, and CTRP5 overexpression markedly attenuated I/R induced cardiomyocytes apoptosis (Figure 2). I/R induced the enhancement of caspase 3 activity, which could be significantly inhibited by CTPR5 overexpression (Figure 2). Furthermore, we also presented that Bax, a pro-apoptosis protein, was significantly up-regulated, but Bcl2, an anti-apoptosis protein, was down-regulated after I/R, however, CTRP5 overexpression successfully inhibited Bax expression but promoted Bcl2 expression (Figures 2F,G). We also analyzed the ATP production in different pathophysiological conditions. I/R induced a significant reduction of ATP in the WT or WT+AAV-GFP group, however, CTRP5 overexpression increased the ATP production compared to the WT+AAV-GFP group (Supplementary Table S2).

### CTRP5 Overexpression Attenuated MI Associated Heart Failure

**FIGURE 3:**
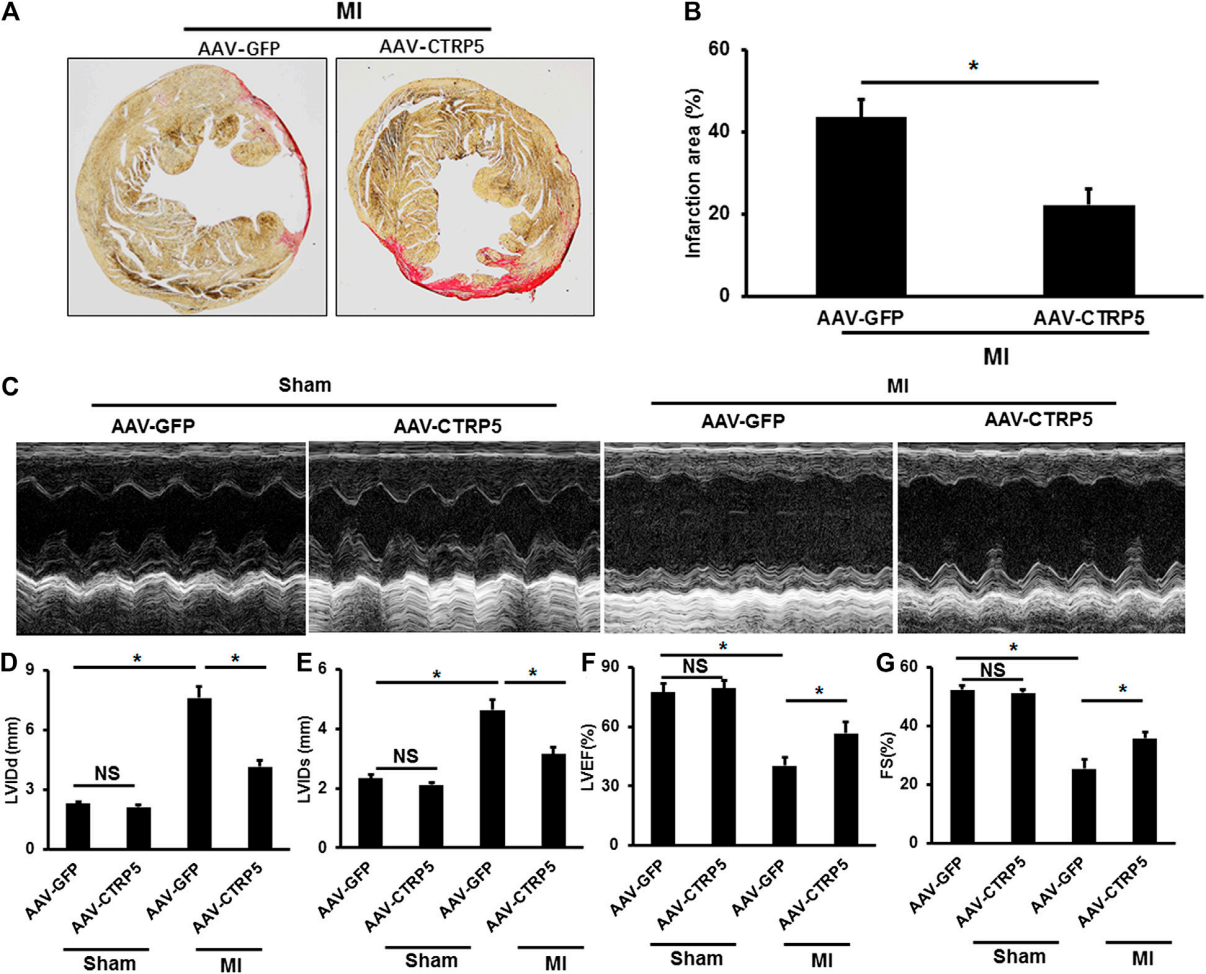
Adeno-associated virus serum type 9 (AAV9) mediated CTRP5 overexpression improved MI-induced cardiac dysfunction and heart failure. **(A,B)** Representative pictures of PSR staining and quantitative analysis of infarction area showed that CTRP5 overexpression reduced infarction area after 21 days of MI surgery (*N* = 6). **(C)** Representative pictures of M-mode echocardiography for assessing cardiac function after at 21st day after MI surgery (*n* = 7–10). Echocardiographic analysis presented that CTRP5 overexpression decreased **(D)** LVIDd and **(E)** LVIDs and increased **(F)** LVEF and **(G)** FS. Data are shown as mean ± SEM. *p < 0.05 represented the difference between the two groups connected by line was statistically significant.

Mice with or without CTRP5 overexpression were subjected to left anterior descending artery ligation to establish an MI associated heart failure model. CTRP5 was continuously overexpressed by AAV9 after 4 weeks of MI (Figure 3). We found that CTRP5 overexpression significantly attenuated MI areas (Figure 3). Figure 3 represented the M mode images of echocardiography and showed a significantly enlarged left ventricular cavity after MI, which could be improved after CTRP5 overexpression. The left ventricular dimensions (LVID; D and LVID’s) were significantly enlarged after MI compared to the control group (Figure 3), which could be significantly decreased after CTRP5 overexpression (Figure 3). The left ejection fraction (LVEF) and shortening fraction (FS) were significantly decreased after MI (Figure 3), but CTRP 5 overexpression significantly improved cardiac functions shown by increased LVEF and FS (Figure 3).

### CTRP5 Overexpression Activated AMP-Activated Protein Kinase Signaling Pathway

**FIGURE 4:**
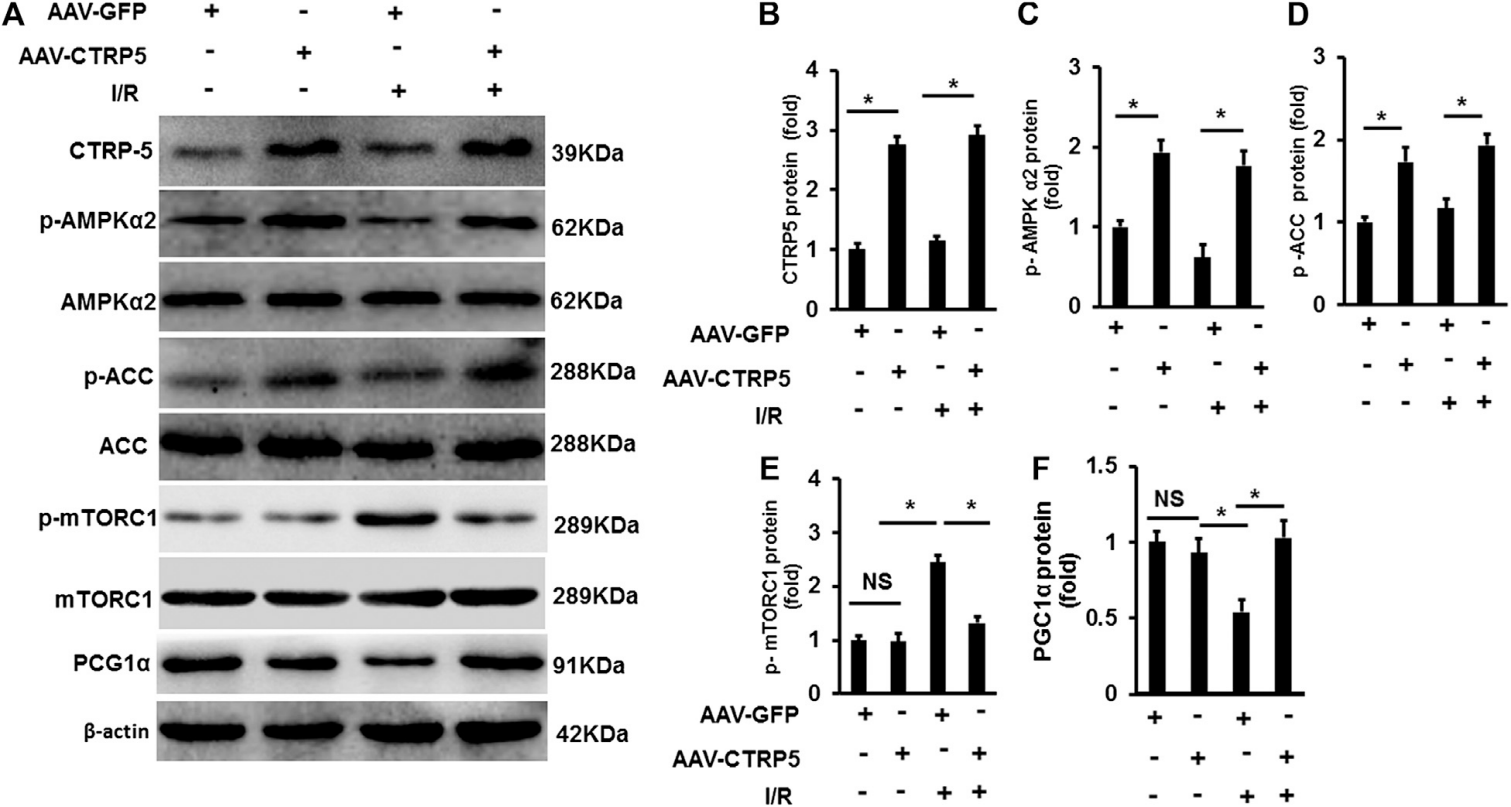
C1q/TNF-related protein 5 (CTRP5) overexpression enhanced AMPK signaling in mouse heart after I/R Mice were administrated with AAV-GFP or AAV-CTRP5 for 3 weeks and then were subjected to sham or I/R surgery. Hearts were harvested for determining the protein levels of **(A)** CTRP5, phosphorylated AMP-activated protein kinase alpha 2 (p-AMPKα2), AMPKα2, phosphorylated acetyl-CoA carboxylase (p-ACC), ACC, p-mTORC1, mTORC1, and PGC1α. Relative quantification of **(B)** CTRP3, **(C)** p-AMPKα2, **(D)** p-ACC, **(E)** p-mTORC1, and **(F)** PGC1α. The relative expression of CTRP3 and PGC1α (normalized to β-actin). The relative expression of p-AMPKα2, p-ACC, and p-mTORC1 was normalized to its corresponding total protein and β-actin respectively. Data are shown as mean ± SEM. *p < 0.05 represented the difference between the two groups connected by line was statistically significant.

AMP-activated protein kinase (AMPK) has been demonstrated to protect against ischemia associated injuries and cellular apoptosis. We examined the activity of AMPK at Thr-172 phosphorylation site in the mouse hearts with or without I/R operation. I/R induced an increase of AMPK phosphorylation (Figure 4), but CTRP5 overexpression enhanced the AMPK phosphorylation both at I/R and sham groups (Figure 4). We also assessed the changes of downstream targets of AMPK in the mouse hearts, including acetyl-CoA carboxylase (ACC), PGC1α, and mTORC1. This study exhibited that CTRP5 overexpression enhanced p-ACC and PGC1α expression, but significantly inhibited mTORC1 phosphorylation (Figures 4A–F).

### CTRP5 Overexpression Attenuated Hypoxia-Reoxygenation Associated Injury in NRCMS

**FIGURE 5:**
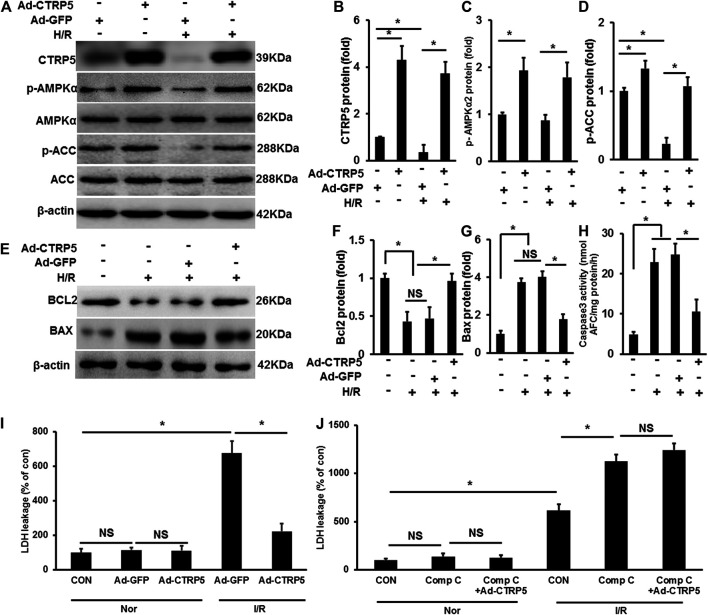
C1q/TNF-related protein 5 (CTRP5) overexpression protected against NRCMs apoptosis Isolated NRCMs were transfected with Ad-GFP or Ad-CTRP5 for 24 h, followed by hypoxia for 24 h firstly and then reoxygenation for 12 h. **(A)** Western-blot was performed for determining protein expression of CTRP5, p-AMPKα2, AMPKα2, p-ACC, ACC, and β-actin. Relative quantification of **(B)** CTRP5, **(C)** p-AMPKα2, and **(D)** p-ACC. **(E–G)** Blots and relative quantification of the apoptosis associated proteins of BCL2 and BAX. **(H)** Caspase 3 activity analysis kit was used to determine cleaved caspase 3 activity. **(I,J)** Lactate dehydrogenase (LDH) Assay Kit was used to detect the LDH leakage, and compound C (Comp C, 10 mm) was used to inhibit AMPK activity. The relative expression of CTRP5, BCL2, and BAX were normalized to β-actin. The relative expression of p-AMPKα2 and p-ACC were normalized to its corresponding total protein and β-actin respectively. Data are shown as mean ± SEM. *p < 0.05 represented the difference between the two groups connected by line was statistically significant.

In isolated NRCMS, CTRP5 overexpression activated p-AMPKα2 and p-ACC at baseline (Figures 5A–D). Hypoxia-Reoxygenation (H/R) induced the down-regulation of p-AMPKα2 and p-ACC and this regulation was blocked by CTRP5 overexpression (Figures 5A–D). We presented that H/R promoted BAX expression and enhanced caspase 3 activity, both of which were demonstrated to contribute to cardiomyocyte apoptosis, but down-regulated the expression of pro-survival protein Bcl_2_ (Figures 5E–H). We also detected the lactate dehydrogenase (LDH) leakage in NRCMs. H/R significantly induced LDH release compared to none in the treatment group, however, CTRP5 overexpression prevented LHD release in H/R treated NRCMs (Figure 5I). Compound C was used to inhibit the AMPK activity *in vitro* experiment. H/R treatment significantly exaggerated the LDH release compared to none in the compound C treated group (Figure 5J). CTRP5 overexpression no longer prevented H/R induced LDH release after compound C mediated AMPK activity inhibition (Figure 5J).

### CTRP5 Overexpression Failed to Protect Against Ischemia/Reperfusion or Myocardial Infarction Associated Heart Injuries After AMPKα2 Knockout

**FIGURE 6:**
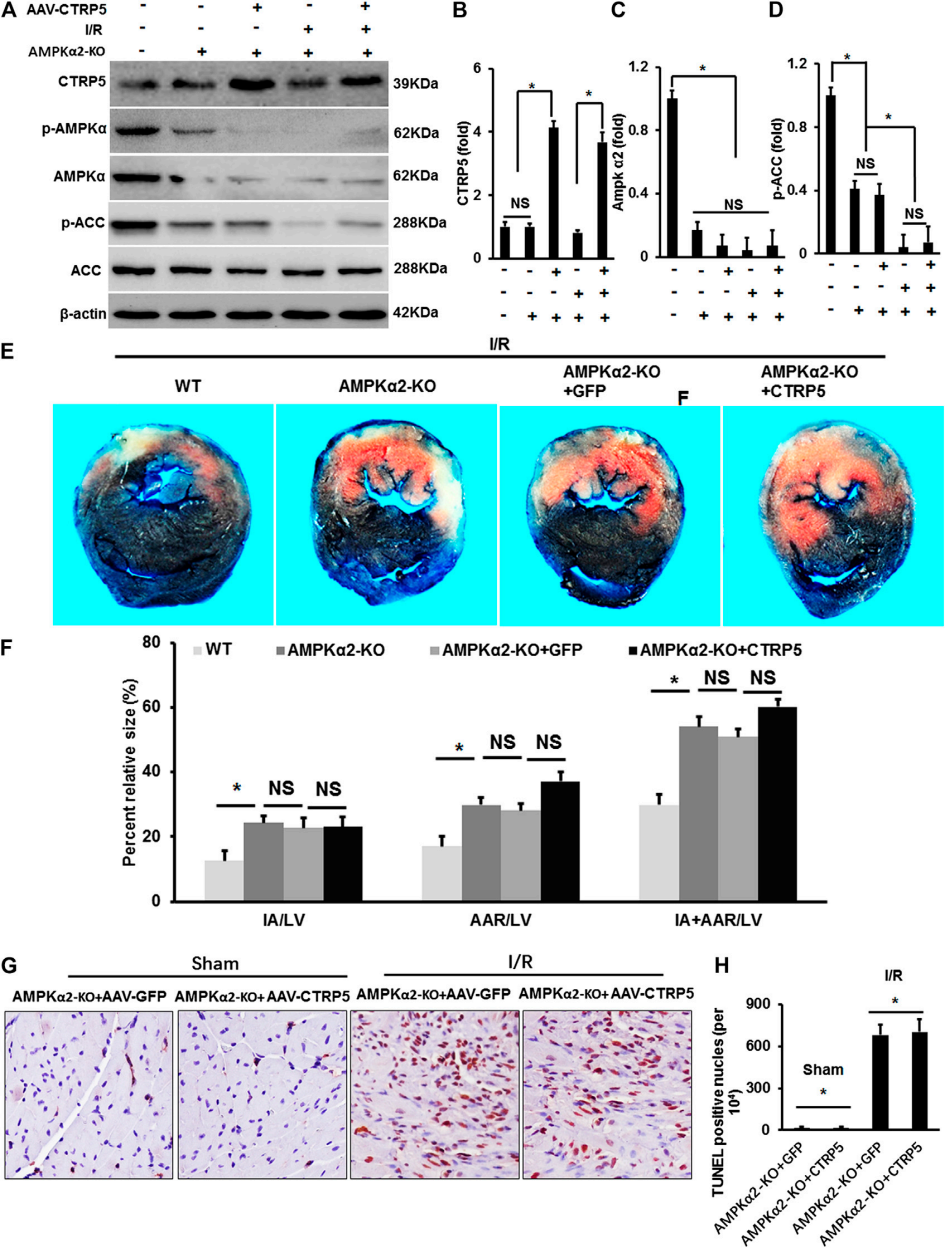
Adeno-associated virus serum type 9 (AAV9) mediated CTRP5 overexpression could not attenuate ischemia/reperfusion associated injuries in AMPKα2-knock out (AMPKα2-KO) mice. **(A)** AMPKα2-KO mice were administrated with an AAV vector to express GFP (AAV-GFP, 4.0 × 10^7^ PFU) or human CTRP5 (AAV-CTRP5, 4.0 × 10^7^ PFU) for 3 weeks and then were subjected to I/R surgery. **(A)** Western-blot was performed for determining protein expression levels of CTRP5, p-AMPKα2, AMPKα2, p-ACC, ACC, and β-actin. Relative quantification of **(B)** CTRP5, **(C)** p-AMPKα2, and **(D)** p-ACC in AMPKα2-KO mouse heart tissue. **(E)** Heart sections were stained with Evans blue and 2,3,5-triphenyltetrazolium chloride (TTC) after I/R and the results indicated that CTRP5 overexpression could not attenuate I/R associated mouse heart injury. **(F)** Quantitative analysis of the infarct area (IA, white)/left ventricle area (IA/LV), area at risk (AAR, red)/LV (AAR/LV) and (IA+AAR)/LV (*N* = 10). **(G,H)** Representative pictures of TUNEL staining and quantitative analysis for apoptotic nuclei (*N* = 10). The relative expression of CTRP5 was normalized to β-actin. The relative expression of p-AMPKα2 and p-ACC were normalized to its corresponding total protein and β-actin respectively. Data are shown as mean ± SEM. *p < 0.05 represented the difference between the two groups connected by line was statistically significant.

AMPKα2 knockout (AMPKα^−/−^) mice were infected with AAV-CTRP5 or AAV-GFP. After 3 weeks of infection, we examined the overexpression of CTRP5 in AMPKα^−/−^ mouse heart, and AMPKα and p-AMPKα were successfully deleted in AMPKα^−/−^ mouse heart (Figures 6A–C). p-AAC was significantly down-regulated and was unable to be activated by CTRP5-overexpression in AMPKα^−/−^ mouse heart (Figure 6). AMPKα^−/−^ mice with or without CTRP5 overexpression were subjected to 60 min of myocardial ischemia and 24 h of reperfusion. As shown in Figures 6E,F, AMPKα2 knock out enlarged the IA/LV, AAR/LV and (IA+AAR)/LV compared to WT subjected to I/R surgery, however, CTRP 5 overexpression showed no effects on IA/LV, AAR/LV and (IA+AAR)/LV in AMPKα^−/−^ mice. The TUNEL staining indicated that I/R induced cardiomyocytes apoptosis, however, CTRP5 overexpression failed to attenuate I/R induced cardiomyocyte apoptosis with AMPKα2 knockout (Figures 6G,H). Furthermore, CTRP5 could no longer recover the ATP production after AMPK α2 knockout (Supplementary Table S3).

**FIGURE 7:**
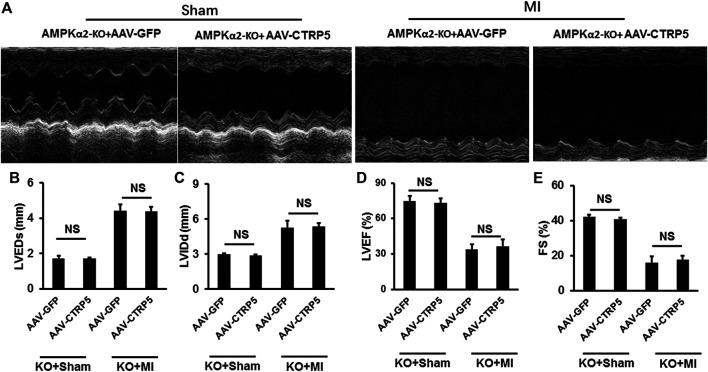
Adeno-associated virus serum type 9 (AAV9) mediated CTRP5 overexpression could not attenuate MI-induced cardiac dysfunction in AMPKα2-KO mice. **(A)** Representative pictures of M-mode echocardiography for assessing cardiac function at the 21st day after MI surgery (*n* = 7–10). Echocardiographic analysis presented that CTRP5 overexpression showed no protective effects on cardiac function, as evidenced by **(B)** LVEDs, **(C)** LVIDd, **(D)** EF, and **(E)** FS values. Data are shown as mean ± SEM. NS indicated no significant difference *p < 0.05 represented the difference between the two groups connected by line was statistically significant.

Figure 7 represented the M mode images of echocardiography and the results showed that MI induced the enlargement of the left ventricular dimensions, which could not be improved by CTRP5 overexpression in AMPKα^−/−^ mouse. In detail, MI induced a significant increase of the LVEDd and LVEDs (Figure 7) and decrease of LVEF and FS (Figure 7) and CTRP5 overexpression showed no significant effects on these parameters in AMPKα^−/−^ mouse.

## Discussion

In this study, we found that cardiomyocytes-specific CTRP5 overexpression protected against I/R and MI associated cardiomyocyte apoptosis and cardiac dysfunction. CTRP5 is a secreted protein and is recognized as a paralog of Adiponectin, but its biological function in heart disease remains unreported. Adiponectin is secreted from adipose tissue and participates in regulating energy metabolism via regulating the AMPK signaling pathway. We tested whether CTRP5 possesses a similar biological function as Adiponectin in regulating ischemia associated heart diseases. This study exhibited that CTRP5 was abundantly secreted in mouse serum as reported by other studies, but we first demonstrated that CTRP5 was also expressed in cardiomyocytes. CTRP5 overexpression could ameliorate ischemia or hypoxia induced cardiomyocyte apoptosis, decrease myocardial infarct size, and improve cardiac function after MI or I/R. However, we observed no obvious differences between CTRP5 overexpression and control groups after I/R or MI operation in AMPKα2^−/−^ mouse. This study demonstrated that CTRP5 could protect mouse hearts from ischemia or hypoxia induced injuries *in vivo* and *in vitro* via the AMPKα2 signaling pathway.

Cardiomyocyte apoptosis and its unrenewable characteristic are the main causes for the occurrence and development of cardiac dysfunction and heart failure induced by ischemia (Del Re et al., 2019). In this study, CTRP5 overexpression significantly alleviated cardiomyocyte apoptosis in an I/R murine model, and also attenuated hypoxia-reoxygenation induced NRCMS apoptosis *in vitro*. Similarly, Yang and Lee (2014) and Yang et al. (2015) reported that treatment with the globular domain of CTRP5 significantly suppressed palmitate induced myocyte apoptosis and reduced the caspase-3 activation. Thus, the ability of CTRP5 to reduce myocardial infarct size after I/R and improve heart failure after MI was partly via reducing cellular apoptosis in the heart.

CTRP5 possesses a homologous domain structure with Adiponectin, which has been demonstrated to protect cardiomyocytes from ischemia induced apoptosis via directly activating the AMPK signaling pathway (Shibata et al., 2005). Transduction with dominant negative AMPK completely blocked the anti-apoptotic effects of Adiponectin in cardiomyocytes challenging serum deprivation and hypoxia-reoxygenation (Shibata et al., 2005). CTRP5 and CTRP9 belong to the same family. Kambara et al. found that circulating CTRP9 overproduction before ischemia could markedly decrease the myocardial infarct size (Kambara et al., 2012). Mechanistically, CTRP9 could promote AMPK phosphorylation at the threonine residue 172 (Kambara et al., 2012), and thus attenuate cellular apoptosis. The silencing of AMPK signaling could completely offset the protective effects of CTRP9 (Kambara et al., 2012). Park (Park et al., 2009) reported that CTRP5 is drastically induced following mitochondrial dysfunction. And it could increase glucose uptake and metabolism for improving mitochondrial function and energy supplement via promoting AMPK phosphorylation (Park et al., 2009). Consistent with previous findings, this study exhibited that CTRP5 overexpression promoted AMPK phosphorylation at threonine residue 172 in ischemic mouse heart tissue and isolated NRCMS exposed to hypoxia-reoxygenation. Moreover, AMPKα2 deficiency completely neutralized the protective effects of CTRP5 overexpression in I/R and MI murine models.

Besides its pro-survival activity, AMPK is also an important energy sensor for modulating glucose and fatty acid metabolism during ischemia-reperfusion and myocardial infarction (Qi and Young, 2015). AMPK phosphorylation could drive glucose uptake by promoting intracellular GLUT4 to the sarcolemma membrane, increase glycolysis by phosphorylating 6-phoshpofructo -2-kinase (PFK2), and inhibit glycogen synthesis by depressing the glycogen synthase activity, which finally resulted in more ATP production (Ma and Li, 2015; Del Re et al., 2019). AMPK activity is also critical to both fatty acid uptake and oxidation. Fatty acid metabolism provides the majority of ATP supplementary in adult cardiomyocytes in normal aerobic condition (Ma and Li, 2015; Del Re et al., 2019). AMPK could decrease the intracellular malonyl-CoA via phosphorylating acetylcoenzyme A carboxylase (ACC) and thus reduce carnitine palmitoyltransferase 1 (CPT-1) inhibition, which could import fatty acyl CoA into the mitochondria and accelerate fatty acid oxidation (Ma and Li, 2015). I/R injury is closely associated with reduced mitochondrial turnover and biogenesis (Qi and Wang, 2020). AMPK mediated peroxisome proliferator-activated receptor gamma coactivator 1 alpha (PGC1α) expression could restore mitochondrial turnover and biogenesis, which contributes to reduced mitochondrial oxidative stress and apoptosis (Qi and Wang, 2020). Previous studies have indicated that AMPK phosphorylation was significantly up-regulated after 2 min of ischemia and reached its peak after 10 min, and then quickly fell back to basal level at the begin of reperfusion (Ma and Li, 2015). Therefore, AMPK phosphorylation has been demonstrated to prevent I/R or MI associated cardiac dysfunction. In this study, we showed direct and precise evidence that CTRP5 could promote AMPK phosphorylation and PGC1α production. Taken together, our study revealed that CTRP5 protected against ischemia associated cardiomyocyte apoptosis and improved mouse cardiac function after I/R or MI through activating AMPK dependent pro-survival pathway.

Some limitations need to be addressed in future investigations. Firstly, the exact mechanism of CTRP5 activating the AMPK phosphorylation has not been shown in this study. However, it owns a homologous domain structure to adiponectin and CTRP9, the regulation of CTRP5 on AMPK phosphorylation was not through AdipoR1 and AipoR2 as adiponectin and CTRP9 (Park et al., 2009; Liu et al., 2019). Secondly, the regulatory mechanism for CTRP5 expression change in ischemic heart disease remains unclear and needs further investigation. Thirdly, clinical observations will be required to clarify the relationships of circulating CTRP5 levels and examining clinical outcomes in patients with ischemic heart diseases.

## Conclusion

In conclusion, CTRP5 overexpression in cardiomyocytes protected against I/R injury and MI associated cardiac dysfunction by activating the AMPKα2 signaling pathway and inhibiting mTORC1 phosphorylation and cardiomyocyte apoptosis. This study furthers understanding of the role and mechanisms of CTRP5 in regulating ischemia associated heart diseases and suggests the potential strategy of targeting CTRP5 in the treatment and prevention of ischemia diseases and related complications.

## Data Availability Statement

The raw data supporting the conclusions of this article will be made available by the authors, without undue reservation.

## Ethics Statement

The animal study was reviewed and approved by the Ethics Committee of the Zhengzhou University.

## Author Contributions

MP and H-BY contributed to the conception and design of the experiments. YL and X-QZ contributed to the acquisition of the data. Y-WX and Y-TZ contributed to the analysis and interpretation of the data. All authors contributed to article preparation.

## Funding

This work was supported by the Scientific and Technological Project of Henan Province (202102310364).

## Conflict of Interest

The authors declare that the research was conducted in the absence of any commercial or financial relationships that could be construed as a potential conflict of interest.
