# Supplementary material for: CTRP5-Overexpression Attenuated Ischemia-Reperfusion Associated Heart Injuries and Improved Infarction Induced Heart Failure
Source: Front Pharmacol. 2020 Dec 22;11:603322. doi: 10.3389/fphar.2020.603322 (PMC7783420; doi:10.3389/fphar.2020.603322)
Supplement: Supplementary file 1 [file datasheet1.doc]

Supplementary Material

# Supplementary Table 1

| Genes | Forward primer | Reverse primer |
| --- | --- | --- |
| IL 1α | ATCAGCAACGTCAAGCAACG | GGTGCTGATCTGGGTTGGAT |
| IL 6 | CACAGAGGATACCACTCCCA | TCCACGATTTCCCAGAGAACA |
| TNF α | ACAAACCACCAAGTGGAGGAG | CTTTGAGATCCATGCCGTTGG |
| ICAM 1 | TGGAGACGCAGAGGACCTTA | CCGCTCAGAAGAACCACCTT |
| VCAM 1 | GCTGCTATTGGCTGTGACTC | GCTCAACACAAGCGTGGATTT |
| GAPDH | CCAATGTGTCCGTCGTGGAT | TCAGATGCCTGCTTCACCAC |

# Interleukin 1α (IL 1α), Interleukin 1 (IL 6), tumor necrosis factor alpha (TNFα), intercellular cell adhesion molecule-1 (ICAM 1), vascular cell adhesion molecule-1 (VCAM-1), glyceraldehyde-3-phosphate dehydrogenase (GAPDH).

# Supplementary Table 2

|  | WT | WT+AAV-GFP | WT+AAV-CTRP5 |  |
| --- | --- | --- | --- | --- |
| Basline | 4.7±0.3 | 4.5±0.3 | 5.1±0.4 | |
| I/R | 2.8±0.2*^ | 2.5±0.3*^ | 4.3±0.3^#^ | |

# Supplementary Table 2 CTRP5 overexpression promoted ATP production in I/R mouse heart

Values (μmol/g wet weight of heart tissue) were presented as the mean ± SEM (n=6). * *p*<0.05 versus WT, # *p*<0.05 versus WT+AAV-GFP, ^ *p*<0.05 versus baseline.

# Supplementary Table 3

|  | WT | AMPKα2-KO | AMPKα2-KO+AAV-GFP | AMPKα2-KO+AAV-CTRP5 |
| --- | --- | --- | --- | --- |
| Basline | 4.6±0.3 | 3.8±0.2 | 3.7±0.4 | 3.8±0.3 |
| I/R | 2.7±0.1*^ | 1.2±0.3*^ | 1.4±0.1^ | 1.3±0.2^ |

**Supplementary Table 3 CTRP5 overexpression could not promote ATP production in I/R mouse heart after AMPK α2 knock out.** Values (μmol/g wet weight of heart tissue) were presented as the mean ± SEM (n=6). * *p*<0.05 versus WT, ^ *p*<0.05 versus baseline.

## Supplementary Figure1


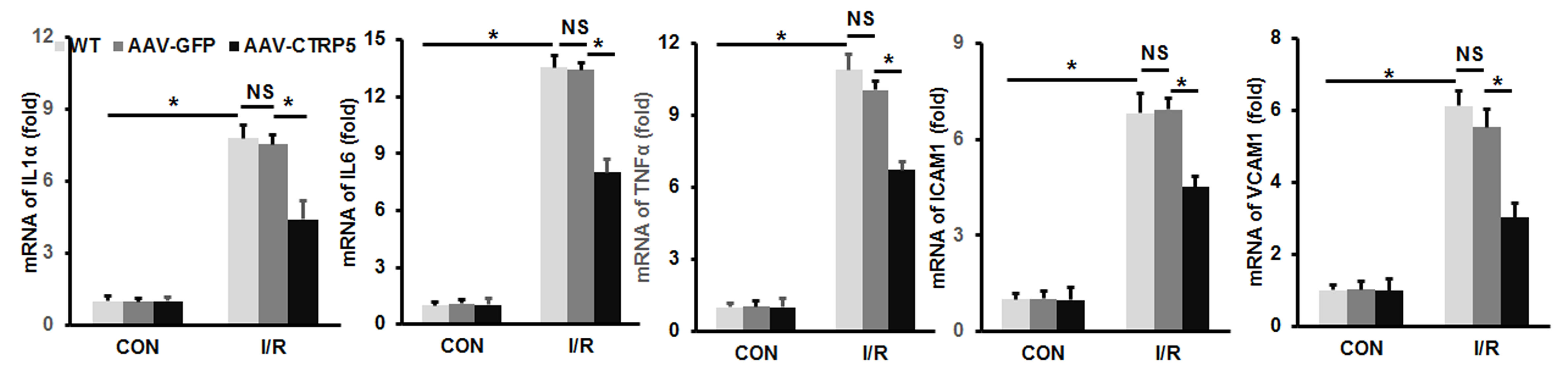


**Supplementary Figure 1.** **CTRP5** **Overexpression prevented I/R induced inflammatory cytokine production** Interleukin 1α (IL 1α), tumor necrosis factor α (TNF α), intercellular cell adhesion molecule-1 (ICAM 1), vascular cell adhesion molecule-1 ( VCAM-1), glyceraldehyde-3-phosphate dehydrogenase (GAPDH) was used for internal reference, and all of these mRNA expression were normalized to GAPDH before the relative quantitative calculation. n=6 * *p<0.05*, compared with indicated group, NS: none significance difference between indicated groups.

## Supplementary Figure2

**
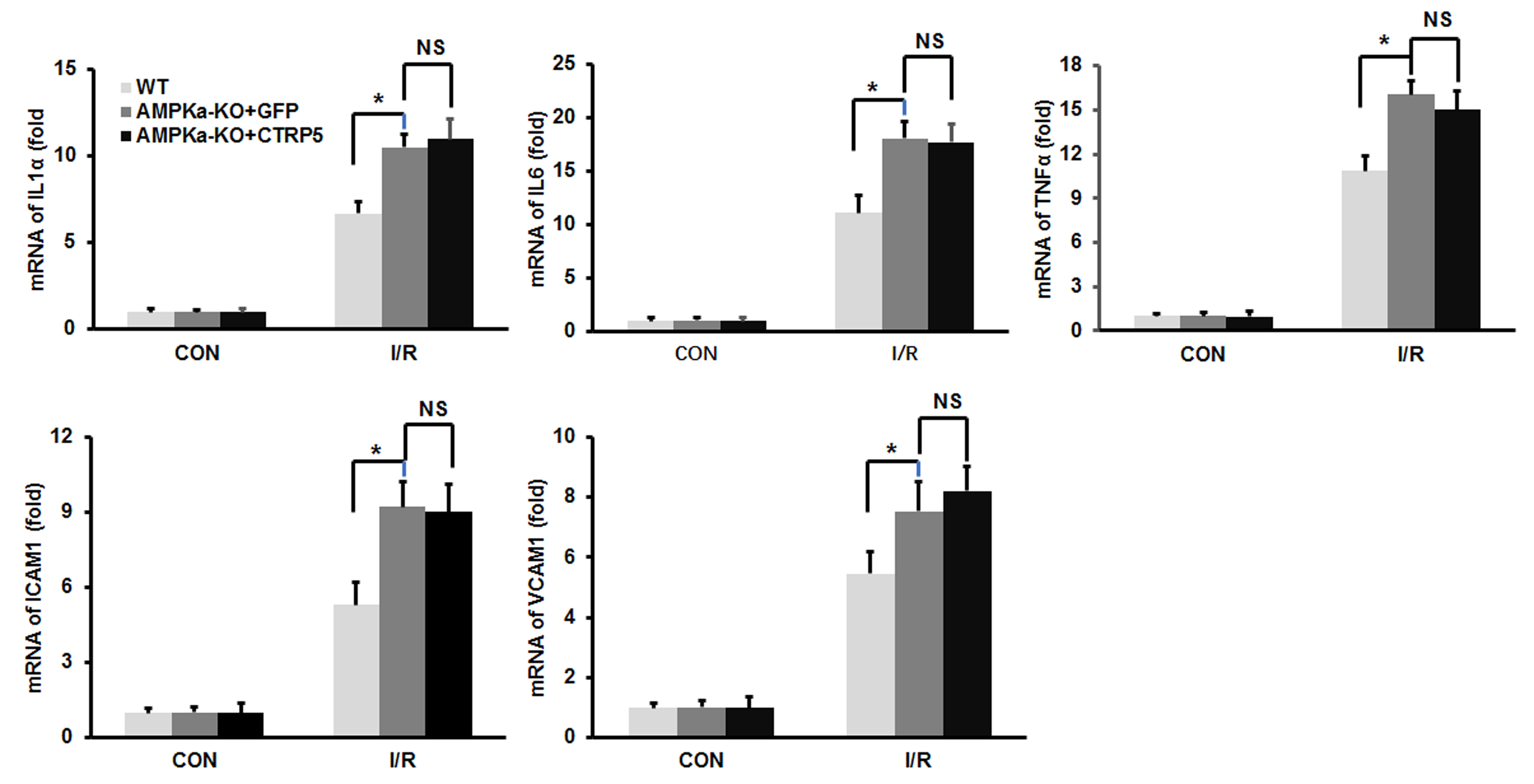
**

**Supplementary Figure 2.** **CTRP5** **Overexpression could not prevent I/R induced inflammatory cytokine production in AMPKα2 knock out mice** Interleukin 1α (IL 1α), tumor necrosis factor α (TNF α), intercellular cell adhesion molecule-1 (ICAM 1), vascular cell adhesion molecule-1 ( VCAM-1), glyceraldehyde-3-phosphate dehydrogenase (GAPDH) was used for internal reference, and all of these mRNA expression were normalized to GAPDH before the relative quantitative calculation. n=6 * *p<0.05*, compared with indicated group, NS: none significance difference between indicated groups.
